# Editorial: Host-pathogen interactions interplay and cancer: understanding mechanisms and beyond

**DOI:** 10.3389/fonc.2023.1202004

**Published:** 2023-04-26

**Authors:** Abdul Arif Khan, Ejaz Ahmad

**Affiliations:** ^1^ Division of Microbiology, ICMR-National AIDS Research Institute, Pune, Maharashtra, India; ^2^ Department of Pathology, University of Michigan Medical School, Ann Arbor, MI, United States

**Keywords:** oncogenesis, microbial pathogenesis, microbes and cancer, anticancer, cancer biology

This editorial summarizes the contributions to the Frontiers Research topic “*Host-Pathogen Interactions Interplay and Cancer: Understanding Mechanisms and Beyond*”, established under the section Molecular and Cellular Oncology of the Frontiers in Oncology.

Role of microbes in cancer is evident through several recent studies. It is apparent that microbes play multifaceted and complex role in the oncogenesis ranging from development of cancer to its prevention. The role of several virus and bacteria are established in the oncogenesis (1), on the other hand several other microbes are under investigation to understand their suspicious role in cancer development, detection and prevention (2, 3). The role of microbiome in cancer is under investigation to understand almost every aspect of carcinogenesis, including cancer development, prevention, diagnosis, response to therapy and treatment. The effects of microbes on the host are mediated through highly regulated and complex host-pathogen interactions contributing to almost every facet of microbial pathogenesis (4). The knowledge about host-pathogen interactions mediated through cancer associated microbes may uncover several caveats about microbial involvement in oncogenesis. Future research investigations may uncover several new insights about the interplay between microbes and cancer through host-pathogen interactions.

This Research Topic encompasses multidisciplinary articles focusing on diverse aspects of microbial involvement in oncogenesis mediated through host-pathogen interactions. Overall, the authors of every manuscript covered different aspects of microbial involvement in cancer development. The research topic consists of article related to basic research using computational approaches along with review articles discussing novel aspects of microbial involvement in cancer development through unique host-pathogen interactions. These manuscripts are ranging from specific role of individual microorganism to collective involvement of microbiome in the cancer development.

The role of hepatitis C virus in hepatocellular carcinoma was discussed with emphasizing the interactions of viral proteins with host cells leading to impairment of normal signaling pathways, inflammation and oxidative stress ultimately contributing to carcinogenesis. In addition, the role of hepatitis C virus in neurological diseases was also discussed as some patients with hepatocellular carcinoma also developed neurological complications. This article tried to gather the details about advancements in the field of hepatitis C virus mediated hepatocellular carcinoma including diagnosis, mechanisms and antiviral therapies related to hepatitis C virus mediated hepatocellular carcinoma (Suhail et al.).

The articles included in the research topic also discussed structural biological insights to the pathogen mediated cancer. The 3D structure of host-pathogen interaction complexes can help to understand the importance of these interactions in microbial pathogenesis including their involvement in pathogen mediated cancer. It also provides an idea about importance of host-pathogen interaction predictions and the gaps in computational approaches for host-pathogen protein-protein interaction prediction. At the end the article discusses about how these approaches can be beneficial in the field of cancer research and provides details for utilization of these approaches in understanding pathogen medicated cancer (Ozdemir and Nussinov).

In addition, the role of tumor microbiome in tumor microenvironment was also discussed in the research topic by the authors. This article focuses on the composition of tumor microenvironment and the studies pertaining to understanding tumor microbiome in different solid tumors. Moreover, this article tries to cover clinical application of microbiome based approaches for cancer management. In conclusion, the article provides comprehensive information about tumor microbiome and its contribution in tumor microenvironment (Ciernikova et al.).

The research article included in Research Topic encompasses novel approaches for drug repurposing using biological interaction data. The authors demonstrated the use of this approach for cervical cancer which is almost exclusively associated with human papillomavirus (HPV) infection. The authors used certain conditions to screen important host proteins from interaction networks. These conditions were (i) differential expression of host proteins, and the author screened transcriptomic datasets using gene expression omnibus (GEO) for identifying differentially expressed genes with cervical cancer. (ii) the relationship of host proteins and cervical cancer was evaluated using statistical significance by adjusted p-value. (iii) Along with that, the authors also screened number of interactions between host and viral proteins and used the rationale that the host targets with more interactions with viral proteins will affect virus more effectively and thus increase the chances of preventing disease progression. In addition, the host target should have low numbers of interaction with human proteins in order to display fewer side effects on the host. (iv) Moreover, the host protein expression level in cervical cancer was taken as another parameter to evaluate is efficacy as a host drug target. Using this approach, the authors identified novel drug candidates and targets for HPV16 and 18 those are majorly involved in the cases of cervical cancer. The approach devised in this manuscript can be used to identify drug candidates for several other infectious diseases including pathogen associated cancer (Kori et al.).

From the application perspective, the manuscripts included in the research topic generate thought provoking space for the readers and generate ideas for using the information for understanding the mechanistic enigma of pathogen associated cancer and the role of host-pathogen interactions in this process. The findings presented in research article can help in the development of novel investigational *in silico, in vitro* and *in vivo* approaches for leveraging translational potential of the findings and their replication in several other diseases including pathogen medicated carcinogenesis.

Altogether, the research article and reviews compiled in this Research Topic provides an important resource for understanding the diverse insights about pathogen mediated cancer and the role of host-pathogen interactions in this process. The review articles highlight the major milestones achieved so far in understanding role of microbes and microbiome in cancer development and using recent approaches to understand this association. On the other hand, the research findings elaborate the strategy for identification of therapeutic targets and drug repurposing for such pathogen mediated cancer using host-pathogen interactions data. Current approaches are generating high throughput host-pathogen interaction data which is being compiled in several dedicated databases and the articles collected in this research topic provides avenues for their utilization in understanding pathogen mediated cancers and their management strategies. We expect our readers deify the noticeable collection of research on cancer related host-pathogen interactions.

## Author contributions

AAK and EA prepared and finalized the manuscript. All authors contributed to the article and approved the submitted version.
